# An angiopoietin-like protein 2 autocrine signaling promotes EMT during pancreatic ductal carcinogenesis

**DOI:** 10.18632/oncotarget.2635

**Published:** 2014-10-24

**Authors:** Carmine Carbone, Geny Piro, Matteo Fassan, Anna Tamburrino, Maria Mihaela Mina, Marco Zanotto, Paul J Chiao, Claudio Bassi, Aldo Scarpa, Giampaolo Tortora, Davide Melisi

**Affiliations:** ^1^ Digestive Molecular Clinical Oncology Research Unit, Università degli studi di Verona, Verona, Italy; ^2^ Laboratory of Oncology and Molecular Therapy, Department of Medicine, Università degli studi di Verona, Verona, Italy; ^3^ ARC-Net Research Centre and Department of Pathology, Diagnostics and Surgery, Università degli studi di Verona, Verona, Italy; ^4^ Department of Molecular and Cellular Oncology, The University of Texas MD Anderson Cancer Center, Houston, TX, USA; ^5^ Medical Oncology Unit, Azienda Ospedaliera Universitaria Integrata, Verona, Italy; ^6^ Pancreas Institute, Azienda Ospedaliera Universitaria Integrata, Verona, Italy

**Keywords:** ANGPTL2, EMT, pancreatic cancer, LILRB2

## Abstract

The identification of the earliest molecular events responsible for the metastatic dissemination of pancreatic ductal adenocarcinoma (PDAC) remains critical for early detection, prevention, and treatment interventions. In this study, we hypothesized that an autocrine signaling between Angiopoietin-like Protein (ANGPTL)2 and its receptor leukocyte immunoglobulin-like receptor B2 (LILRB2) might be responsible for the epithelial-to-mesenchymal transition (EMT) and, the early metastatic behavior of cells in pancreatic preneoplastic lesions.

We demonstrated that the sequential activation of KRAS, expression of HER2 and silencing of p16/p14 are sufficient to progressively and significantly increase the secretion of ANGPTL2, and the expression of LILRB2. Silencing the expression of ANGPTL2 reverted EMT and reduced migration in these cell lines. Blocking ANGPTL2 receptor LILRB2 in KRAS, and KRAS/HER2/p16p14shRNA LILRB2- expressing cells reduced ANGPTL2-induced cell proliferation and invasion. An increasingly significant overexpression of ANGPTL2 was observed in in a series of 68 different human PanIN and 27 PDAC lesions if compared with normal pancreatic parenchyma.

These findings showed that the autocrine signaling of ANGPTL2 and its receptor LILRB2 plays key roles in sustaining EMT and the early metastatic behavior of cells in pancreatic preneoplastic lesions supporting the potential role of ANGPTL2 for early detection, metastasis prevention, and treatment in PDAC.

## INTRODUCTION

Pancreatic ductal adenocarcinoma (PDAC) is a deadly disease [1, 2]. The poor prognosis for patients with PDAC could be mainly attributed to its aggressive course, the limited efficacy of available systemic treatments, and, in particular, to the invariable metastatic behavior demonstrated along the progression of the disease [3]. The majority of patients are, indeed, diagnosed with metastatic disease, and only 15-20% of patients are eligible for initial resection [4]. In patients who undergo surgery and post-operative therapy, metastatic relapse remains common and no more than 20% of patients achieve long-term survival [5]. Thus, the identification of the earliest molecular events responsible for the metastatic dissemination of PDAC remains critical for early detection, prevention, and treatment interventions.

Morphological and genetic changes associated with pancreatic carcinogenesis have been well described [6]. Pancreatic intraepithelial neoplasia (PanINs) is the most common precursor to invasive PDAC. Genetic analyses of these lesions showed that they harbor many of the same genetic alterations identified in invasive PDAC. In particular, activating mutations in *KRAS* are among the earliest genetic alterations in pancreatic carcinogenesis, with codon 12 mutations already detectable in 92.0% of PanIN-1A lesions [7]. These alterations are commonly followed by inactivating mutations in the *p16/CDKN2A* tumor suppressor gene in PanIN-1/2 lesions [7], and in *TP53* and *DPC4* in the latest stages of carcinogenesis [8].

Conversely, the molecular mechanisms that promote the metastatic spread of PDAC are less clear [9]. Previous genetic studies applying high-throughput genetic analyses to paired primary and metastatic PDAC tissues proposed that metastasis is a late event in the clonal evolution of this disease [10]. More recent studies using a mathematical modeling approach with radiological and pathological data on PDAC patients who underwent autopsy revealed that all patients are expected to harbor cells that are capable of metastasis in the primary tumor at the time of diagnosis, even when the size of the primary tumor is small [11]. Further evidences supporting the model that metastasis is an early event in pancreatic carcinogenesis have been provided by using a genetically engineered murine model of PDAC in which the pancreatic epithelial cells could be tracked during tumor progression through the expression of YFP allele into the *KRas* plus *p53* or *p16* mutant background. In this model, even low-grade PanINs showed evidence of cells that have crossed the basement membrane, migrated from the glandular epithelium into the surrounding tissue and circulatory system, and seeded the liver prior to PDAC formation. This behavior was associated with an early epithelial-to-mesenchymal transition (EMT) in the premalignant lesions [12].

Angiopoietin-like Protein (ANGPTL)2 is a member of a family of seven secreted glycoproteins that are structurally related to Tie-2 receptor ligands angiopoietins [13], but do not bind to either Tie-2 or the homologous Tie-1 receptor [14]. ANGPTL2 is an important adipocyte-derived mediator of chronic inflammation in obesity, and in its related systemic insulin resistance [15]. Increasing expression levels of ANGPTL2 were measured during carcinogenesis in a chemically induced skin squamous cell carcinoma model [16]. We have recently demonstrated that ANGPTL2 is among the proinflammatory factors that are overexpressed and induce EMT in PDAC cells with acquired resistance to anti-VEGF treatment [17]. Importantly, the human leukocyte immunoglobulin-like receptor B2 (LILRB2) has been recently identified as the receptor for ANGPTLs. A deficiency in the intracellular signaling of its mouse orthologue paired immunoglobulin-like receptor (PIRB) resulted in increased differentiation of leukemia cells [18].

In this present study, we hypothesized that an autocrine signaling between ANGPTL2 and its receptor LILRB2 might be responsible for the early EMT and, in turn, the tumor progression in a model of multistep accumulation of genetic lesions in pancreatic ductal cells. Thus, silencing the expression of ANGPTL2 might modulate the early metastatic behavior of cells in pancreatic premalignant lesions.

## RESULTS

### Serial expression of activated KRAS, HER2, and p16/p14 silencing induces EMT features in HPDE and HPNE cells

In order to demonstrate our hypothesis, we used two *in vitro* immortalized and non-tumorigenic pancreatic epithelial cell lines, the human papilloma virus (E6E7)-immortalized human pancreatic ductal epithelial (HPDE) and the hTERT-immortalized human pancreatic ductal epithelial nestin-expressing cell line (HPNE). These *in vitro* experimental cell transformation model systems consisted in the sequential and stable expression of activated KRAS, HER2, and shRNA sequences to knock down the expression of p16/p14 [19].

**Figure 1:**
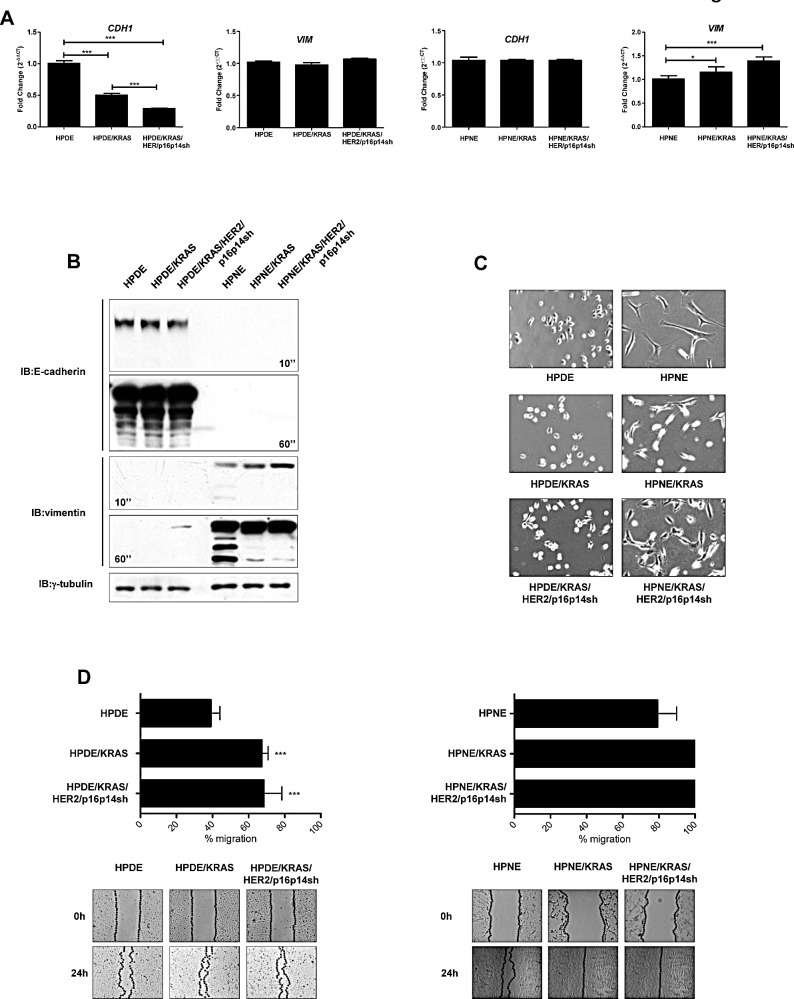
Features of EMT in the different steps of pancreatic carcinogenesis A, results of quantitative real-time PCR analysis of E-cadherin (*CDH1*) and vimentin (*VIM*) genes presented as the fold change in RNA expression between the gene of interest and β-actin. The mean values and SEM from 3 independent experiments conducted in quadruplicate are shown. ***, P < 0.001; *, P<0.05, by one-way ANOVA and Dunnett's multiple comparison post test. B, western blot analysis of EMT markers E-cadherin and vimentin. γ-tubulin was used as loading control. C, light-microscopic phenotype in HPDE, HPDE/KRAS, HPDE/KRAS/HER2/p16p14shRNA and HPNE, HPNE/KRAS, HPNE/KRAS/HER2/p16p14shRNA cell lines. D, levels of cell migration in HPDE and HPNE in vitro cell transformation model systems. Results are presented as percentages of the total distances between the wound edges enclosed by cells. The mean values and SEM from 3 independent experiments done in quadruplicate are shown. ***, P < 0.001 by one-way ANOVA and Dunnett's multiple comparison post test. Photographs of the wound area were taken by using phase-contrast microscopy immediately (0) and 24 hours after the incision.

By using these models, we initially studied the features of EMT in the different steps of the pancreatic progression. Whereas the expression of the mesenchymal marker vimentin was not regulated in the HPDE/KRAS and in the HPDE/KRAS/HER2/p16p14shRNA cell lines when compared with the HPDE normal control, the expression of the epithelial marker E-cadherin was progressively and significantly reduced in these cell lines representing different steps of development in pancreatic carcinogenesis. Conversely, the expression of the mesenchymal marker vimentin was progressively and significantly increased in HPNE/KRAS and in the HPNE/KRAS/HER2/p16p14shRNA cell lines when compared with the HPNE normal control cells (*P*<0.001; Figure 1, A). Consistently, western blot analysis confirmed the higher expression of vimentin in KRAS and KRAS/HER2/p16p14shRNA cells if compared with normal control cells (Figure 1, B).

Whereas HPNE cells demonstrated already a mesenchymal-like appearance, HPDE/KRAS and HPDE/KRAS/HER2/p16p14shRNA cells had an increasingly different light-microscopic appearance, including a more spindle-shaped morphology, if compared with HPDE control cells (Figure 1, C).

During malignant progression, it has been proposed that carcinoma cells undergoing an EMT not only lose their epithelial characteristics, but also acquire migratory properties that are critical for tumor dissemination [20]. In a wound closure assay, HPDE and HPNE /KRAS and /KRAS/HER2/p16p14shRNA cell lines had significantly higher migration and invasive rates than did their respective control cells (*P*<0.001; Figure 1, D and Figure S1). Moreover, in order to justify the lower invasive properties of HPDE in comparison with HPNE cell models, we performed a zymography assay (Figure S1), demonstrating an higher MMP2 activity in HPNE than in HPDE cell model system accordingly to their more mesenchymal and aggressive features.

These results indicate that activation of KRAS, expression of HER2 and silencing of p16/p14 are sufficient to induce EMT in human pancreatic ductal epithelial normal cells.

### Secreted ANGPTL2 induces increasing EMT features in HPDE and HPNE cells based on LILRB2 expression

**Figure 2:**
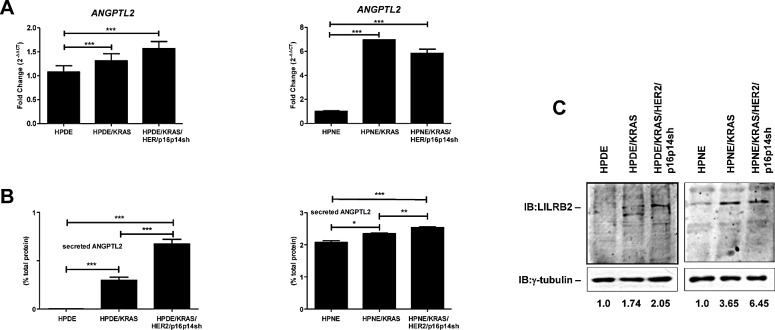
Expression and secretion of ANGPTL2 and its receptor LILRB2 in the different steps of pancreatic carcinogenesis A, results of quantitative real-time PCR analysis of ANGPTL2 gene presented as the fold change in RNA expression between the gene of interest and β-actin. The mean values and SEM from 3 independent experiments conducted in quadruplicate are shown. ***, P < 0.001, by one-way ANOVA and Dunnett's multiple comparison post test. B, ELISA analysis of the secretion of ANGPTL2 in the conditional medium of HPDE and HPNE cell lines. Results are presented as percentages of the total protein extract from the respective secreting cells. The mean values and SEM from 3 independent experiments conducted in quadruplicate are shown. ***, P < 0.001; *, P < 0,05, by one-way ANOVA and Dunnett's multiple comparison post test. C, Western blot analysis for the expression of LILRB2 in HPDE and HPNE *in vitro* cell transformation model systems. Relative LILRB2 protein levels were quantified by the ratio between LILRB2 and γ-tubulin, which were arbitrarily set at 1.0 for control cells.

To study the role of ANGPTL2 in the progression of pancreatic preneoplastic lesions, we measured its expression and secretion in the HPDE and HPNE /KRAS and /KRAS/HER2/p16p14shRNA and in their respective control cells. We observed that the different cell lines along these experimental cell transformation model systems showed increasing expression levels of ANGPTL2 (*P*<0.001; Figure 2, A). Whereas, the HPDE and HPNE normal control cells showed undetectable or low ANGPTL2 secretion levels, the serial expression of activated KRAS, HER2, and p16/p14 shRNA induced a significantly increased secretion of ANGPTL2 (*P*<0.001; Figure 2, B).

To sustain the existence of an ANGPTL2 autocrine signaling, we measured the expression of the ANGPTL2 receptor LILRB2 in these cell lines. We demonstrated an overexpression of LILRB2 in the /KRAS and in the /KRAS/HER2/p16p14shRNA expressing cell lines when compared with the normal control cells (Figure 2, C).

**Figure 3:**
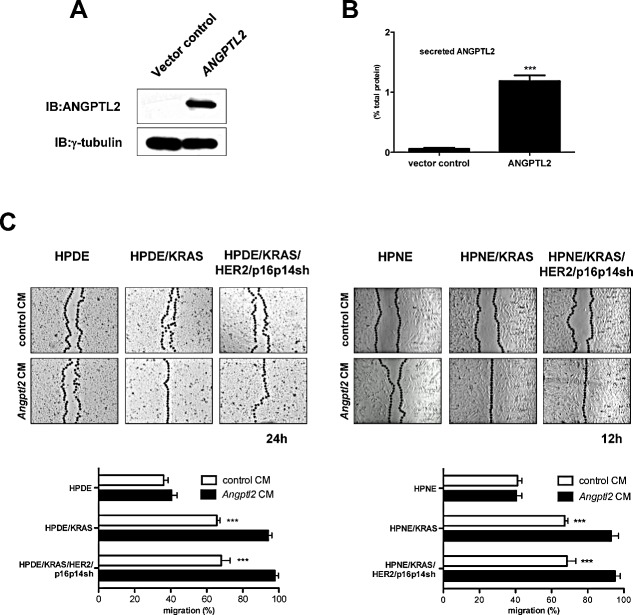
Exogenous ANGPTL2 induces EMT features in HPDE and HPNE cell lines based on LILRB2 expression A, Western blot analysis for the expression of ANGPTL2 in HEK293T cells transfected with hANGPTL2-RFP-2A-Puro (ANGPTL2) or control vector. B, ELISA analysis of the secretion of ANGPTL2 in the conditional medium from HEK293T cell lines transfected with hANGPTL2-RFP-2A-Puro (ANGPTL2) or control vector. Results are presented as percentages of the total protein extract from the respective secreting cells. The mean values and SEM from 3 independent experiments conducted in quadruplicate are shown. ***, P < 0.01 by two-tailed unpaired Student's t tests. C, levels of cell migration in HPDE, HPDE/KRAS, HPDE/KRAS/HER2/p16p14shRNA and HPNE, HPNE/KRAS, HPNE/KRAS/HER2/p16p14shRNA cell lines if cultured with conditional medium from HEK293T cell lines transfected with hANGPTL2-RFP-2A-Puro (*ANGPTL2* CM) or control vector (control CM). Results are presented as percentages of the total distances between the wound edges enclosed by cells. The mean values and SEM from 3 independent experiments done in quadruplicate are shown. ***, P < 0.001 by two-tailed unpaired Student's t tests. Photographs of the wound area were taken by using phase-contrast microscopy at indicated time point after the incision.

In order to study the role of secreted ANGPTL2 on the malignant features of cells during pancreatic carcinogenesis, we expressed ANGPTL2 gene in HEK293T cells (Figure 3, A). We measured a significantly increased secretion of ANGPTL2 protein in the conditional medium from these cells when compared with HEK293T vector control cells (*P*<0.001; Figure 3, B). Thus, we cultured HPDE and HPNE normal control cells, and /KRAS and /KRAS/HER2/p16p14shRNA expressing cell lines with the conditional medium from ANGPTL2-expressing HEK293T or HEK293T control cells. In a wound closure assay, the migratory properties of HPDE and HPNE cell lines were not affected if cultured with the conditional medium from ANGPTL2-expressing HEK293T. On the contrary, HPDE or HPNE cells expressing KRAS, or KRAS/HER2/p16p14shRNA demonstrated a significantly higher migration rate if cultured with the conditional medium from ANGPTL2-expressing HEK293T than did if cultured with the conditional medium from control HEK293T cells, closing the distance between the wound edges after 24 hours and 12 hours respectively (*P*<0.001; Figure 3, C and S2).

### Silencing the expression of ANGPTL2 reverts EMT in HPDE and HPNE *in vitro* cell transformation system cell lines

**Figure 4:**
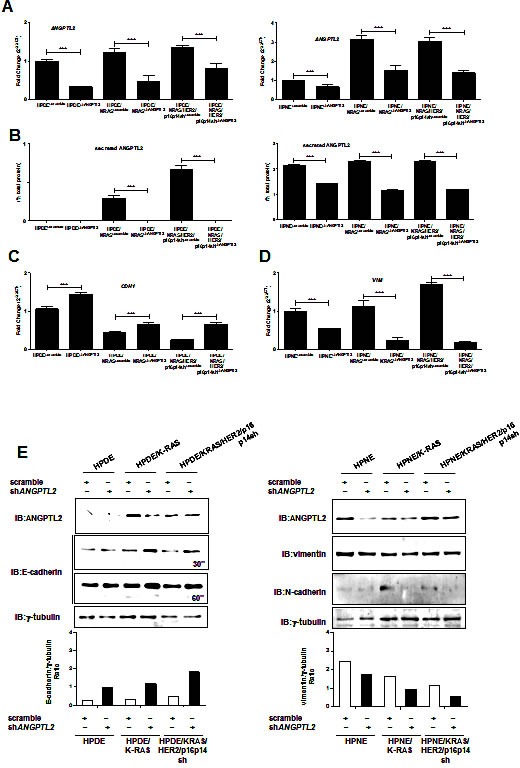
Silencing the expression of ANGPTL2 reverts EMT in HPDE and HPNE /KRAS, /KRAS/HER2/p16p14shRNA expressing cell lines A, results of quantitative real-time PCR analysis of ANGPTL2 gene expression in HPDE and HPNE, /KRAS, /KRAS/HER2/p16p14shRNA cell lines expressing shRNA sequences to knock down the expression of ANGPTL2 or scramble sequence as control. Results are presented as the fold change in RNA expression between the gene of interest and β-actin. The mean values and SEM from 3 independent experiments conducted in quadruplicate are shown. ***, P < 0.001 by two-tailed unpaired Student's t tests. B, ELISA analysis of the secretion of ANGPTL2 in the conditional medium of HPDE and HPNE, /KRAS, /KRAS/HER2/p16p14shRNA cell lines expressing shRNA sequences to knock down the expression of ANGPTL2 or scramble sequence as control. Results are presented as percentages of the total protein extract from the respective secreting cells. The mean values and SEM from 3 independent experiments conducted in quadruplicate are shown. ***, P < 0.001 by two-tailed unpaired Student's t tests. C, results of quantitative real-time PCR analysis of *CDH1* gene expression in HPDE, HPDE/KRAS and HPDE/KRAS/HER2/p16p14shRNA cell lines expressing shRNA sequences to knock down the expression of ANGPTL2 or scramble sequence as control. D, results of quantitative real-time PCR analysis of *vimentin* gene expression in HPNE, HPNE/KRAS and HPNE/KRAS/HER2/p16p14shRNA cell lines expressing shRNA sequences to knock down the expression of ANGPTL2 or scramble sequence as control. Results are presented as the fold change in RNA expression between the gene of interest and β-actin. The mean values and SEM from 3 independent experiments conducted in quadruplicate are shown. ***, P < 0.001 by two-tailed unpaired Student's t tests. E, Western blot analysis for the expression of E-cadherin and vimentin in HPDE and HPNE, /KRAS and /KRAS/HER2/p16p14shRNA cell lines expressing shRNA sequences to knock down the expression of ANGPTL2 or scramble sequence as control. The relative protein quantification was reported as ratio with γ-tubulin protein expression levels.

To test our hypothesis that ANGPTL2 might be responsible for the early EMT in pancreatic carcinogenesis, we used an shRNA sequence to knock down the expression of ANGPTL2 in both *in vitro* HPDE and HPNE cell transformation systems. With this approach, we were able to significantly reduce the expression (*P*<0.001; Figure 4, A) and the secretion (*P*<0.001; Figure 4, B) of ANGPTL2 in all of the cell lines.

To determine the effect of silencing ANGPTL2 on the EMT features in these *in vitro* cell line models of pancreatic carcinogenesis, we initially evaluated the expression of the epithelial marker gene E-cadherin in ANGPTL2- knockdown and control cell lines. We demonstrated that the expression of E-cadherin gene was significantly upregulated in ANGPTL2 knockdown cell lines compared with the respective controls (*P*<0.001; Figure 4, C). Consistently, the HPDE/KRAS, and HPDE/KRAS/HER2/p16p14shRNA cell lines expressing shRNA sequences to knock down the expression of ANGPTL2 had significantly higher levels of E-cadherin than did their respective controls (Figure 4, E). Interestingly, the expression of vimentin gene was significantly downregulated in ANGPTL2 knockdown HPNE cell lines compared with their respective controls (*P*<0.001; Figure 4, D). Consistently, the HPNE/KRAS, and HPNE/KRAS/HER2/p16p14shRNA cell lines expressing shRNA sequences to knock down the expression of ANGPTL2 had significantly lower levels of mesenchymal proteins vimentin and N-cadherin than did their respective controls (Figure 4, E).

**Figure 5:**
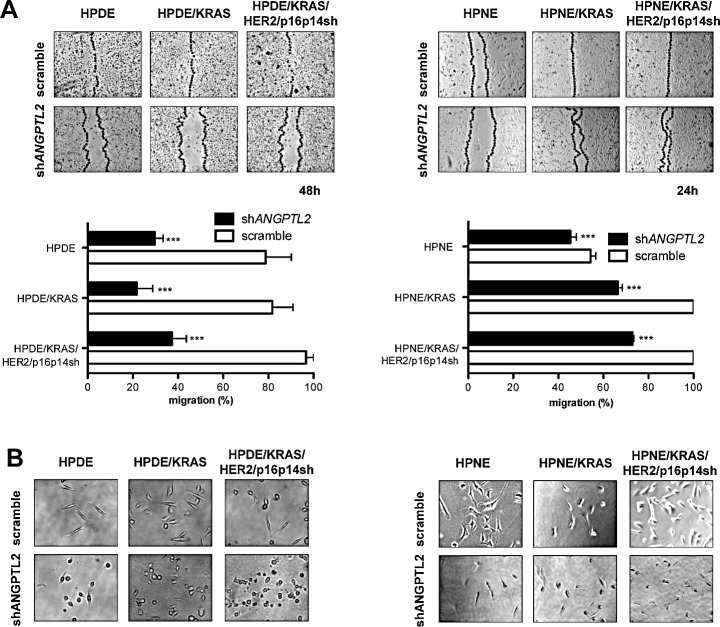
Silencing the expression of ANGPTL2 modulates migratory properties in HPDE and HPNE /KRAS, /KRAS/HER2/p16p14shRNA cell lines A, levels of cell migration in HPDE and HPNE /KRAS, /KRAS/HER2/p16p14shRNA cells expressing shRNA sequences to knock down the expression of ANGPTL2 or scramble sequence as control. Results are presented as percentages of the total distances between the wound edges enclosed by cells. The mean values and SEM from 3 independent experiments done in quadruplicate are shown. ***, P < 0.001 by two-tailed unpaired Student's t tests. Photographs of the wound area were taken by using phase-contrast microscopy immediately at indicated time point after the incision. B, light-microscopic phenotype of HPDE and HPNE /KRAS, /KRAS/HER2/p16p14shRNA cells expressing shRNA sequences to knock down the expression of ANGPTL2 or scramble sequence as control, growing in an equal volume culture medium and Matrigel.

Subsequently, we measured the migratory properties of ANGPTL2 knockdown and control cell lines in a wound closure assay. After 48 hours, when even the poorly migrating HPDE cells were able to cover the distance between the wound edges, HPDE, HPDE/KRAS, and HPDE/KRAS/HER2/p16p14shRNA cell lines expressing ANGPTL2 shRNA sequences demonstrated a strong reduction in their migratory properties if compared with their respective control cell lines (*P*<0.001; Figure 5, A). Consistently, HPNE, HPNE/KRAS, and HPNE/KRAS/HER2/p16p14shRNA cell lines expressing ANGPTL2 shRNA sequences demonstrated a strong reduction in their migratory properties after 24 hours if compared with their respective control cell lines (*P*<0.001; Figure 5, A). Moreover, ANGPTL2 knockdown cells showed a markedly different light-microscopic appearance when cultured in Matrigel, including a more rounded cell morphology, if compared with their respective control cell lines (Figure 5, B).

Taken together, these data demonstrate that autocrine ANGPTL2 is an essential mediator of the EMT and, in turn, the early metastatic behavior in our pancreatic premalignant lesions *in vitro* models.

### ANGPTL2 receptor LILRB2 blocking antibody affected cell proliferation and invasion of *in vitro* cell transformation model systems HPDE and HPNE

In order to study the role of secreted ANGPTL2 on the proliferative and invasive features of cells during pancreatic carcinogenesis, we cultured normal control cells, KRAS and KRAS/HER2/p16p14shRNA expressing cell lines of both HPDE and HPNE model systems with the conditional medium from ANGPTL2-expressing HEK293T and control HEK293T cells.

**Figure 6:**
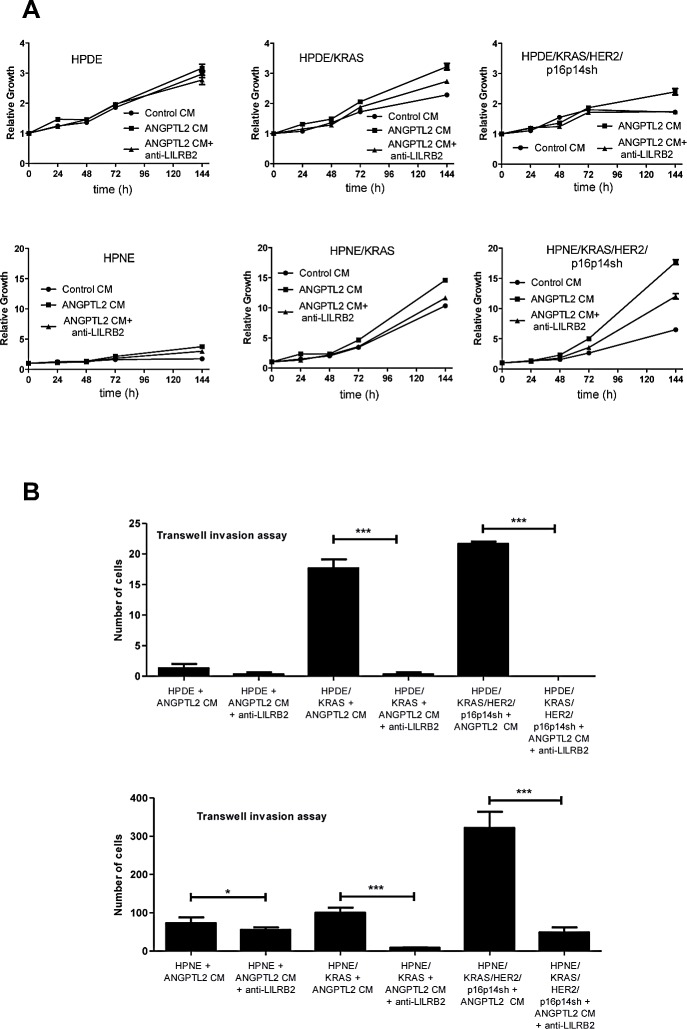
LILRB2 blocking antibody affects cell proliferation and invasion of in vitro cell transformation model systems HPDE and HPNE A, Cell proliferation assay of HPDE and HPNE normal control cells, and /KRAS and /KRAS/HER2/p16p14shRNA cell lines cultured with the conditional medium from ANGPTL2-expressing HEK293T or HEK293T control cells, with or without anti-LILRB2 blocking antibody pre-treatment. The mean values and SEM from 3 independent experiments done in quadruplicate are shown. B, transwell invasion assay of HPDE and HPNE normal control cells, and /KRAS and /KRAS/HER2/p16p14shRNA expressing cell lines cultured with the conditional medium from ANGPTL2-expressing HEK293T or HEK293T control cells with or without anti-LILRB2 blocking antibody pre-treatment. Three invasion chambers were used for treated and untreated group. The values obtained were calculated by averaging the total number of the cells from three filters. All experiments were performed in triplicates. ***, P < 0.001; *, P < 0,05, by one-way ANOVA and Dunnett's multiple comparison post test.

To sustain the existence of an LILRB2/ANGPTL2 autocrine signaling, we blocked the binding of ANGPTL2 receptor LILRB2 to its ligand by using a monoclonal antibody directed against the extracellular portion of LILRB2. Because of their minimal expression of LILRB2 (Figure 2, C), the proliferation rate of HPDE and HPNE control cells was not affected by the exogenous ANGPTL2 contained in the conditional medium from the ANGPTL2-expressing HEK293T cells. In contrast, we measured a significantly increased proliferation rate of HPDE/KRAS and HPNE/KRAS cells, and in particular of the HPDE/KRAS/HER2/p16p14shRNA and HPNE/KRAS/HER2/p16p14shRNA cells if cultured with the conditional medium from ANGPTL2-expressing HEK293T when compared with the conditional medium from control HEK293T cells (*P*<0.001; Figure 6, A). Interestingly, anti-LILRB2 blocking antibody pretreatment of /KRAS and /KRAS/HER2/p16p14shRNA expressing HPDE and HPNE *in vitro* cell transformation model systems, significantly reduced ANGPTL2-induced cell proliferation according to their higher expression of LILRB2. Consistently, the effect on the invasiveness of KRAS and KRAS/HER2/p16p14shRNA expressing HPDE and HPNE *in vitro* cell transformation model systems treated with the conditional medium from the ANGPTL2-expressing HEK293T cells was completely rescued by the pre-treatment of the cells with anti-LILRB2 blocking antibody (Figure 6,B).

These results provide a strong evidence that ANGPTL2-induced cell proliferation and invasion in our pancreatic premalignant lesions *in vitro* models involve the binding of ANGPTL2 to the LILRB2 receptor.

### ANGPTL2 expression in human pancreatic premalignant lesions

In order to determine whether our *in vitro* findings could be verified in PDAC patients, we analyzed a series of sequential lesions (68 different PanINs and 27 PDAC) from 95 surgically resected patients for the expression of ANGPTL2 (Table 1).

**Table 1:** Clinical and histological characteristics from 95 patients with pancreatic cancers

Characteristic	N°	%
**Age, years**		
Median	62,5	
Range	40-81	
Male sex	49/46	51,6
**T Stage**		
T3	90	94,73
T4	5	5,27
**Nodal stage**		
N0	15	15,78
N+	80	84,22
**cM I**		
M0	93	97,89
M+	2	2,11
**Grade**		
G2	59	62,1
G3	36	37,9
**Considered lesions**		
Normal	14	14,73
PanIN I	14	14,73
PanIN II	21	22,1
PanIN III	19	20
PDAC	27	28,44

**Figure 7:**
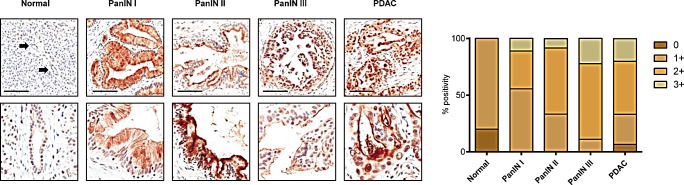
Representative images of ANGPTL2 expression in normal pancreatic parenchyma, PanIN lesions and PDAC A significant overexpression was observed among preneoplastic and neoplastic lesions (Original magnifications 10x and 20x; bars 100 um). ANGPTL2 immunostaining was semiquantified using a four-tier scoring system based on intensity of staining (0= negative; 1= faint/weak; 2= moderate; 3= strong). Mann-Whitney test p<0.0001.

In normal pancreatic parenchyma, ANGPTL2 was moderately expressed in a canalicular pattern of staining in pancreatic acini, whereas a faint/weak luminal and nuclear immunoreaction was observed in duct epithelia. Conversely, a significant overexpression was observed in preneoplastic and neoplastic lesions. PanINs-1 showed a moderate/strong cytoplasmic immunoreaction, with a concurrent luminal component in PanINs-2 cases. Most PanIN-3 and PDAC lesions showed a diffuse ANGPTL2 immunoreaction (Figure 7, Mann-Whitney test p<0.0001).

## DISCUSSION

In the present study, we demonstrated that the secretion of ANGPTL2 and the expression of its receptor LILRB2 are progressively increased in a model of multistep pancreatic carcinogenesis. Silencing the expression of ANGPTL2 modulated the EMT and, in turn, the early metastatic behavior of cells in pancreatic premalignant lesions. Consistently, an increasing overexpression of ANGPTL2 was observed in a series of human pancreatic preneoplastic and neoplastic lesions if compared with normal pancreatic parenchyma.

Previous studies, mainly from the group of Oike and colleagues, provided evidences that ANGPTL2 could contribute to carcinogenesis [16, 21]. In a chemically induced skin squamous cell carcinoma model, transgenic mice constitutively expressing ANGPTL2 under the keratinocyte-specific promoter K14 exhibited accelerated skin carcinogenesis, more mesenchymal tumors, and increased metastases. Conversely, carcinogenesis, EMT, and metastasis were markedly reduced in ANGPTL2 knockout mice [16]. In these models, the chronic inflammation sustained by the constitutive overexpression of ANGPTL2 in the preneoplastic cells as well as in the tumor microenvironment accelerated chemically-induced skin carcinogenesis through increased oxidative stress and decreased expression of the DNA mismatch repair enzyme Msh2 [21]. Our study contributes to the field by defining for the first time the role of ANGPTL2 in PDAC development and early metastatic behavior. Importantly, although PDAC is known to commonly arise in an inflammatory microenviroment [3], our results indicated that in pancreatic ductal carcinogenesis ANGPTL2 is directly secreted by cells in the preneoplastic lesions in an increasing manner along with the accumulation of the most common and early genetic lesions. The increased expression of the ANGPTL2 receptor LILRB2 in HPDE and HPNE /KRAS, and HPDE/KRAS/HER2/p16p14shRNA cell lines if compared with respective controls, the parallel response of these cell lines to exogenous ANGPTL2, and the effect of inhibiting ANGPTL2/LILRB2 binding by blocking anti-LILRB2 monoclonal antibody suggested the existence of an autocrine loop between LILRB2 and its ligand ANGPTL2.

Different studies demonstrated an association between ANGPTL2 expression and the metastatic potential in fully tumorigenic cell lines. In human lung cancer cell lines, constitutive overexpression of ANGPTL2 increased *in vitro* motility and invasive capacity, and accelerated metastasis and shortened mice survival in *in vivo* xenograft models. Accordingly, patients with lung cancer showing elevated expression of ANGPTL2 in cells within the primary tumor were associated with a reduced disease-free survival after surgical resection [22]. More recently, silencing the expression of ANGPTL2 in osteosarcoma cell lines delayed the occurrence of lung metastases when injected in nude mice by reducing the expression of MMP9 [23]. Our results provide the first evidence that silencing the expression of ANGPTL2 could modulate the EMT and, in turn, the early metastatic behavior of preneoplastic cells in the earliest phases of pancreatic ductal carcinogenesis, at a stage that seems to be crucial for the common metastatic dissemination observed in PDAC [12]. These results significantly indicate a potential role for ANGPTL2 as a novel marker for PDAC early detection, and as a target for metastasis chemoprevention.

This study, however, had some limitations. In order to define whether the modulation of EMT and, in turn, of the migratory properties of preneoplastic cell line models by silencing the expression of ANGPTL2 would indeed translate in a reduced incidence of metastasis, an evaluation of the metastatic potential of HPDE and HPNE, /KRAS, and /KRAS/HER2/p16p14shRNA cell lines expressing ANGPTL2 shRNA sequences and of their respective control cell lines should have been performed in an orthotopic *in vivo* model in nude mice. However, as they represent the earliest phases of pancreatic ductal carcinogenesis, these cell lines demonstrated a very low incidence of tumor development when injected in murine models [24]. Thus, an *in vivo* experiment able to significantly discriminate difference in metastasis incidence among these cell lines should have been unethically over dimensioned. In this regard, we corroborated the clinical relevance of our findings for PDAC patients by demonstrating for the first time an increasingly higher and diffuse expression of ANGPTL2 in PanIN-1, -2, and -3, and in PDAC lesions when compared with normal duct epithelia.

In conclusion, this study demonstrates that the autocrine signaling of ANGPTL2 and its receptor LILRB2 plays key roles in sustaining EMT and the early metastatic behavior of cells in pancreatic preneoplastic lesions, thus supporting the potential role of ANGPTL2 for early detection, metastasis prevention, and treatment in PDAC patients.

## MATERIALS AND METHODS

### Cell Lines and Reagents

Human papillomavirus type 16 early gene 6 and 7-immortalized (HPDE) and hTERT-immortalized human pancreatic ductal epithelial nestin-expressing cell line (HPNE) nontumorigenic human pancreatic ductal epithelial cells stably expressing activated *KRAS* (HPDE/KRAS), *HER2*, and shRNA sequences to knock down the expression of p16/p14 (HPDE/KRAS/HER2/p16p14sh) have been previously described in [19, 25]. The HPDE were routinely cultured in keratinocyte serum-free medium supplemented by epidermal growth factor and bovine pituitary extract (Life Technologies, Inc., Grand Island, NY). The HPNE were grown in medium D and DMEM containing one volume of M3 Base F culture medium (InCell Corpor., San Antonio USA), three volumes of glucose free DMEM, 10% FBS5.5mM glucose, 10ng/ml EGF, and 50μg/ml gentamycin. HEK293T cells (Thermo Scientific, MA, USA) were maintained in Dulbecco's Modified Eagle's Media supplemented with 10% heat-inactivated FBS, 20 mmol/L HEPES (pH 7.4), penicillin (100 UI/mL), streptomycin (100 mg/mL), and 4 mmol/L glutamine (ICN Biomedicals Ltd.) in a humidified atmosphere of 95% air and 5% CO2 at 37°C. Secreted ANGPTL2 in conditional medium was measured by ELISA assay (CSB-E13881h Cusabio, P.R. China).

### Reverse transcription-PCR and Real-Time PCR

RNA was isolated by Trizol reagent as per manufacturer's instructions (Invitrogen, Carlsbad, CA, USA). Reverse transcription-PCR (RT-PCR) assay was performed accordingly with the High capacity cDNA reverse transcription kit (Applied Biosystems, Foster City, CA, USA). The mRNA expression of ANGPTL2, CDH1, and Vimentin was quantified by using a SYBR green based real-time PCR analysis and the ABI Prism 7900 HT Sequence Detection System (Applied Biosystems, Foster City, CA, USA). Each gene was tested in each cell line in four replicates and three independent experiments were performed. To quantify the relative changes in gene expression, the 2^−ΔΔCT^ method was used and reactions were normalized to endogenous control gene β-actin expression levels [26].

### Protein extraction and western blotting

Western blot analyses were performed as previously described in [27]. Briefly, HPDE and HPNE cell lines were washed twice with cold phosphate-buffered saline and lysed at 4°C into radioimmunoprecipitation assay buffer (50 mM Tris HCl [pH 8], 150 mM NaCl, 1% Nonidet P-40, 0.5% sodium deoxycholate, and 0.1% sodium dodecyl sulfate). Each lysate (20 μg of protein) was separated by 8% sodium dodecyl sulfate-polyacrylamide gel electrophoresis and probed (1:1000) with mouse monoclonal antibodies against ANGPTL2, CDH1 (Ab-Cam, Cambridge, UK), LILRB2, γ-tubulin (Santa Cruz Biotechnology, Santa Cruz, CA, USA) or Vimentin (Dako Denmark A/S). Immunoreactive proteins were visualized with Lumi-Light western blotting substrate (Roche, Indianapolis, IN) according to the manufacturer's instructions.

### ANGPTL2 overexpression in HEK293T cells

HEK293T cells were seeded onto 6-well plates (Corning, Tewksbury MA, USA) at a density of 5×10^4^ cells/cm^2^, cultured for 24 hours, and grown to confluence. Cells were then transfected by pLenti-GIII-CMV-hANGPTL2-RFP-2A-Puro or vector control (Abm Inc., Richmond, BC Canada). Twenty-four hours after transfection, the culture medium was resplenished, and conditioned for additional 48 hours.

### Cell proliferation assay

HPDE and HPNE *in vitro* cell transformation model systems were seeded at a density of 1.0 × 10^3^ cells/well in 96-well plates. After 24 hours, these cell lines were overlaid with conditional medium from ANGPTL2-overexpressing HEK293T or HEK293T control cells. Cell proliferation was measured at 48, 72 and 96 hours by using the sulforhodamine B assay according to manufacturer's instructions.

### Silencing the expression of ANGPTL2

Retroviral vector pGFP-V-RS shRNA and relative scramble control (Origene, Rockville, MD, USA) were used to transfect our models. HPDE and HPNE cell lines alone or expressing KRAS, and Kras/HER2/p16p14sh were seeded onto 6-well plates (Corning, Tewksbury MA, USA) at a density of 5×10^4^ cells/cm^2^, cultured for 24 hours, and transfected with vector containing ANGPTL2 shRNA or scramble sequence by using TransIT®-LT1 Transfection Reagent (Origene, Rockville, MD, USA) according to manufacturer's instructions.

### Wound healing, transwell invasion and phenotypic assay

Wound healing assay was performed as previously described in [28]. Briefly, HPDE and HPNE *in vitro* cell transformation model systems were seeded at 90% of confluence in 100mm cell culture dishes. After 24 hours, a straight scratch was made using a pipette tip to simulate a wound. The cells were washed gently with cold phosphate-buffered saline (PBS1X) and rinsed with fresh medium. Photographs of at least five different points were taken immediately and after 24 hours of culture.

The phenotype changes in HPDE and HPNE cells were determined by separately seeding 2 × 10^4^ cells in a 50 μL drop of serum free DMEM culture medium mixed with an equal volume of Matrigel. After 48 hours, photographs were taken under a Leica DMIL-Led microscope equipped with a Leica EC3 camera (Leica, Wetzlar, Germany).

*In vitro* invasion assay were performed using 24-well transwell unit with polycarbonate filters (Corning Costar, Cambridge, USA). HPDE and HPNE cells were suspended at density of 5×10^5^/ml in culture medium and then placed in the upper part of transwell. Meanwhile, conditional medium from HEK293T cells expressing ANGPTL2 or control were added at the lower wells of the chambers. LILRB2 treated cells were pretreated for 4 hours with anti LILRB2 antibody (MAB 2078 R&D Systems Inc., Minneapolis, USA). Cells were incubated for 24hours, fixed with ethanol and stained with 0,05% Crystal violet for 30 min. Cells in the upper chamber were removed with cotton swab. Cells that invaded through the Matrigel (Matrigel^TM^ Basement Membrane Matrix, BD Biosciences, USA) to the underside of the filters were counted and picture taken under a Leica DMIL-Led microscope equipped with a Leica EC3 camera (Leica, Wetzlar, Germany). Three invasion chambers were used for treated and untreated group. The values obtained were calculated by averaging the total number of the cells from three filters. All experiments were performed in triplicates.

### Immunohistochemistry of human lesions

A retrospective series of 68 formalin-fixed paraffin-embedded (FFPE) pancreatic intraepithelial neoplasia (PanIN) and 27 PDAC lesions from 95 surgically treated patients were retrieved from the ARC-Net biobank at Verona University Hospital. PanIN lesions were classified according to WHO 2010, and graded as low (PanIN I; 14 lesions), intermediate (PanIN II; 21 lesions), and high grade (PanIN III; 19 lesions). Non-tumor pancreatic parenchyma adjacent to the lesions was considered for scoring. These materials have been collected under Program 853 protocol 298CE 15/02/02 and revised Program 1885 protocol 52438 23/11/2010, approved by the Verona University Hospital ethics committee. The protocols include informed consent from the patient.

Immunohistochemical stainings for ANGPTL2 (AB36014) were obtained on 4-μm-thick FFPE sections using an automated instrument (Bond-maX, Menarini). Sections were lightly counterstained with hematoxylin. Appropriate positive and negative controls were run concurrently. Slides were scored by two pathologists (MF, AS) and a consensus score was reached. ANGPTL2 immunostaining was semiquantified using a four-tier scoring system based on intensity of staining (0= negative; 1= faint/weak; 2= moderate; 3= strong). Subcellular localization of the immunoreaction was annotated.

### Statistical Analysis

All results were expressed as the SEM interval for at least three independent experiments performed in triplicate. All of the statistical analyses were performed using the GraphPad Prism software program (version 4.0c; GraphPad Software, La Jolla, CA).

## SUPPLEMENTARY MATERIALS, FIGURES


