# Supplementary material for: An angiopoietin-like protein 2 autocrine signaling promotes EMT during pancreatic ductal carcinogenesis
Source: Oncotarget. 2014 Oct 24;6(15):13822–34. doi: 10.18632/oncotarget.2635 (PMC4537052; doi:10.18632/oncotarget.2635)
Supplement: Supplementary file 1 [file oncotarget-06-13822-s001.pdf]

An Angiopoietin-like Protei 2 autocrine signaling promotes EMT during pancreatic ductal carcinogenesis

Supplementary Material

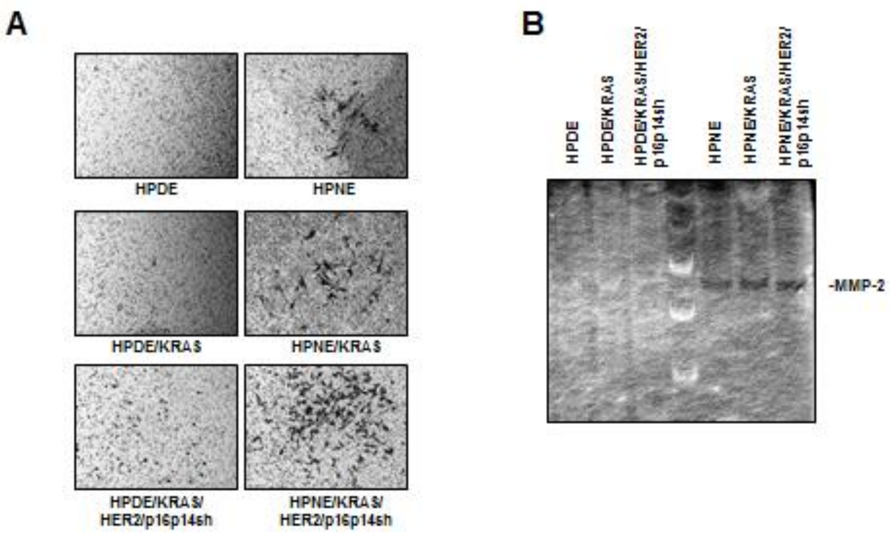

Figure supplementary S1. Features of invasiveness in the different steps of pancreatic carcinogenesis. A, light-microscopic photographs of experiments of Transwell invasion assays. The results are presented as single picture of representative well for each cell line. B, Gelatin zymography, the data shows gelatinase activity of MMP-2 in cellular extracts from HPDE and HPNE in vitro cell transformation model systems.

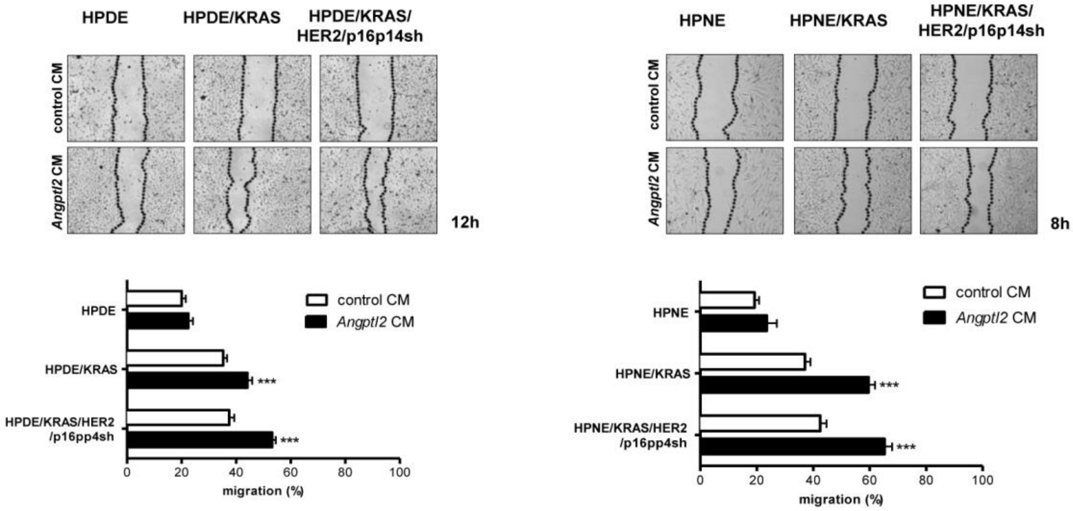

Figure S2. Exogenous ANGPTL2 induces EMT features in HPDE and HPNE cell lines based on LILRB2 expression. Levels of cell migration in HPDE, HPDE/KRAS, HPDE/KRAS/HER2/p16p14shRNA and HPNE, HPNE/KRAS, HPNE/KRAS/HER2/p16p14shRNA cell lines if cultured with conditional medium from HEK293T cell lines transfected with hANGPTL2-RFP-2A-Puro (*ANGPTL2* CM) or vector control (control CM). Results are presented as percentages of the total distances between the wound edges enclosed by cells. The mean values and SEM from 3 independent experiments done in quadruplicate are shown. ***, $P < 0.001$ by two-tailed unpaired Student's t tests. Photographs of the wound area were taken by using phase-contrast microscopy at indicated time after the incision.

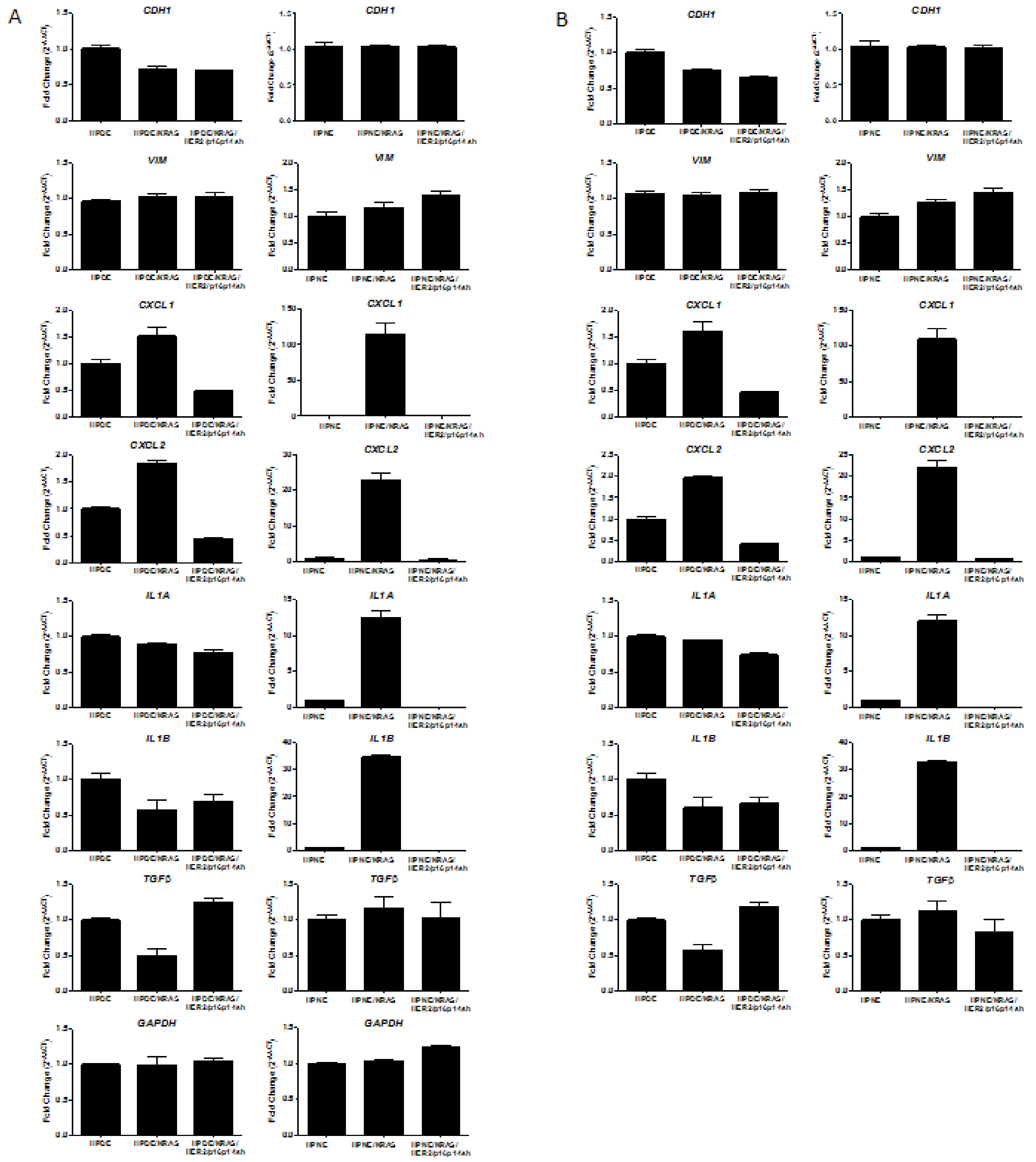

Figure S3. Results of quantitative real time PCR analysis of E-Cadherin, Vimentin, CXCL1, CXCL2. IL1A, IL1B, TGFb and GAPDH genes in HPDE and HPNE cell lines. **A**, The results are presented as fold change in RNA expression between the gene of interest and b-actin. The mean values and SEM from 3 independent experiments conducted in quadruplicate are shown. **B,** The results are presented as fold change in RNA expression between the gene of interest and GAPDH. The mean values and SEM from 3 independent experiments conducted in quadruplicate are shown.
